# Correction: Hopefully devoted to Q: targeting glutamine addiction in cancer

**DOI:** 10.1038/s41416-019-0432-7

**Published:** 2019-03-14

**Authors:** Emma R. Still, Mariia O. Yuneva

**Affiliations:** Oncogenes and Tumour Metabolism Laboratory, The Francis Crick Institute, 1 Midland Road, London, NW1 1AT UK

**Keywords:** Risk factors, Oncology

**Correction to:** *British Journal of Cancer* (2017) **116**:1375–1381; 10.1038/bjc.2017.113; http://www.bjcancer.com; published online: 25 April 2017

This article was originally published under a CC BY NC SA License, but has now been made available under a CC BY 4.0 License.

